# Expanding Pertussis Epidemiology in 6 Latin America Countries through the Latin American Pertussis Project

**DOI:** 10.3201/eid2313.170457

**Published:** 2017-12

**Authors:** Veronica A. Pinell-McNamara, Anna M. Acosta, Maria Cristina Pedreira, Ana F. Carvalho, Lucia Pawloski, Maria Lucia Tondella, Elizabeth Briere

**Affiliations:** Centers for Disease Control and Prevention, Atlanta, Georgia, USA (V.A. Pinell-McNamara, A.M. Acosta, L. Pawloski, M.L. Tondella, E. Briere);; Pan American Health Organization, Washington, DC, USA (M.C. Pedreira);; Sabin Vaccine Institute, Washington (A.F. Carvalho)

**Keywords:** pertussis, whooping cough, respiratory infections, *Bordetella pertussis*, toxoid, Latin America, epidemiological monitoring, surveillance, monitoring, immunologic, sentinel surveillance, pertussis vaccines, diphtheria-tetanus-acellular pertussis vaccines, PCR, serology, CDC, Centers for Disease Control and Prevention, Pan American Health Organization, PAHO, Sabin Vaccine Institute, global health security, Global Health Security Agenda, bacteria

## Abstract

The Latin American Pertussis Project (LAPP), established in 2009, is a collaboration between the Centers for Disease Control and Prevention, Pan American Health Organization, Sabin Vaccine Institute, and the ministries of health of 6 countries in Latin America. The project goal is to expand understanding of pertussis epidemiology in Latin America to inform strategies for control and prevention. Here we describe LAPP structure and activities. After an initial surveillance evaluation, LAPP activities are tailored to individual country needs. LAPP activities align with Global Health Security Agenda priorities and have focused on expanding laboratory diagnostic capacity, implementing a laboratory quality control and quality assurance program, and providing epidemiologic support to strengthen reporting of pertussis surveillance data. Lessons learned include that ongoing mentoring is key to the successful adoption of new technologies and that sustainability of laboratory diagnostics requires a regional commitment to procure reagents and related supplies.

Pertussis, also known as whooping cough, is one of the most poorly controlled vaccine-preventable diseases in the world. The bacterium *Bordetella pertussis* causes the disease, which is endemic worldwide and associated with cyclical increases every 2–5 years (*1*). The disease is typically more severe and associated with more complications in and deaths of infants <1 year of age, particularly those <6 months of age (*1*,*2*). Despite the widespread availability of pertussis vaccines and high vaccination coverage rates, pertussis continues to be a leading cause of death among children (*2*). A recent study modeling pertussis incidence and death estimated that in 2014, there were 24.1 million cases and 160,700 deaths worldwide among children <5 years of age (*3*). Although these findings emphasize the importance of pertussis as a cause of childhood deaths, the estimates are limited by lack of reliable surveillance data and diagnostic capacity (*4*).

The number of pertussis cases reported in the Americas region overall had declined from the early 1980s until the early 2000s, but several countries, including the United States and some countries in Latin America, observed increases in pertussis cases, and outbreaks of pertussis, during that period (*5*–*8*). Given the transmissibility of pertussis and global interconnectivity, such outbreaks can represent a public health threat. Since 2002, many Latin America countries have reported increases in the number of pertussis cases, including Argentina, Brazil, Chile, Colombia, Panama, and Mexico (*5**–**7**,**9**–**13*). However, estimation of the effects of pertussis in Latin America is complicated by the lack of published data on pertussis deaths, country-specific differences in case definitions, and variability of diagnostic tests available (*6*,*9*). In addition, reported pertussis incidence and case fatality rates vary widely among countries in Latin America, despite similar vaccination schedules and coverage (*6*). This difference may be caused partly by areas of suboptimal vaccination coverage within countries, as well as differences in case management, surveillance infrastructure, and case identification by healthcare providers (*8**,**9**,**14**,**15*). The recent increase of reported pertussis and the varied incidence among Latin America countries highlight the need to reinforce surveillance reporting and diagnostic capacity across the region (*5*,*7**,**8*).

Worldwide, diagnosis of pertussis is challenging because the symptoms may resemble those of other respiratory diseases, and the accuracy of available laboratory diagnostics depends on both the timing (Figure 1) and quality of specimen collection (*16*,*17*). Diagnostics recommended by the World Health Organization (WHO) include culture and PCR of nasopharyngeal specimens and serologic testing (*16**–**19*). Direct fluorescent antibody assay is not recommended because it has low sensitivity and specificity for *B. pertussis* (*16**,**17**,**20*); however, it is used in some parts of Latin America (*6*). Because no single pertussis diagnostic assay is optimal for detection of infection at all stages of disease, a complementary testing strategy (i.e., a combination of culture, PCR, and serologic testing) may maximize the surveillance system’s potential for case confirmation (*16*,*17*). However, multiple diagnostics for pertussis are not used or widely available in Latin America, in part because of lack of technical training and limited access to reagents and supplies.

**Figure 1:**
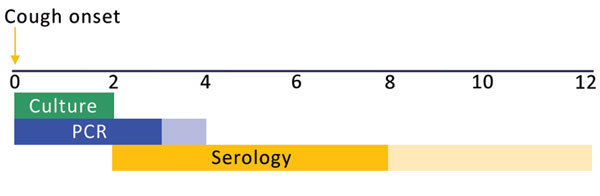
Optimal timing for diagnostic testing for pertussis, in weeks. Dark colors indicate optimal timing window; lighter colors indicate that tests may provide accurate results during these periods.

Strong epidemiologic and laboratory surveillance are crucial to rapidly identifying and controlling pertussis outbreaks and assessing the effects of disease control measures, as well as to monitor changes in pertussis epidemiology and the evolution of the organism (*21*). Throughout the nations of Latin America, reporting pertussis is mandatory, and surveillance systems adhere to WHO surveillance recommendations; however, the countries may have differing case definition criteria (*6**,**22*). Surveillance programs in Latin America face similar barriers to those reported in other regions (*23*), including lack of awareness of the disease, lack of a regional standard case definition, and limited laboratory capacity (*5*,*6*,*8*,*9*). Pertussis reporting in the region tends to focus on cases among hospitalized infants or young children, and cases are often confirmed by clinical criteria only; these factors may lead to underestimation or overestimation of disease prevalence (*6*,*8**,**24*).

In 2009, the Pan American Health Organization (PAHO) Technical Advisory Group on Vaccine Preventable Diseases identified a need for improved epidemiologic information for pertussis to inform vaccination policies and surveillance recommendations (*25*). In this context, the Latin American Pertussis Project (LAPP) was established in 2009 to expand the understanding of pertussis epidemiology in the region by strengthening both laboratory diagnostic capacity and epidemiologic surveillance in selected countries.

## LAPP

LAPP is a collaborative effort between the Centers for Disease Control and Prevention (CDC), PAHO, the Sabin Vaccine Institute (Sabin), and ministries of health (MOHs) of participating countries in Latin America. CDC provides technical support of epidemiology and laboratory diagnostics to partners; PAHO provides expertise on immunizations and coordination with the MOHs; and Sabin provides overall funding and project management, as well as logistical support and feedback for project activities. In each country, the MOH committed national-level public health personnel, including staff from both the pertussis surveillance department and the national reference laboratory (NRL), to participate in LAPP activities.

**Figure 2:**
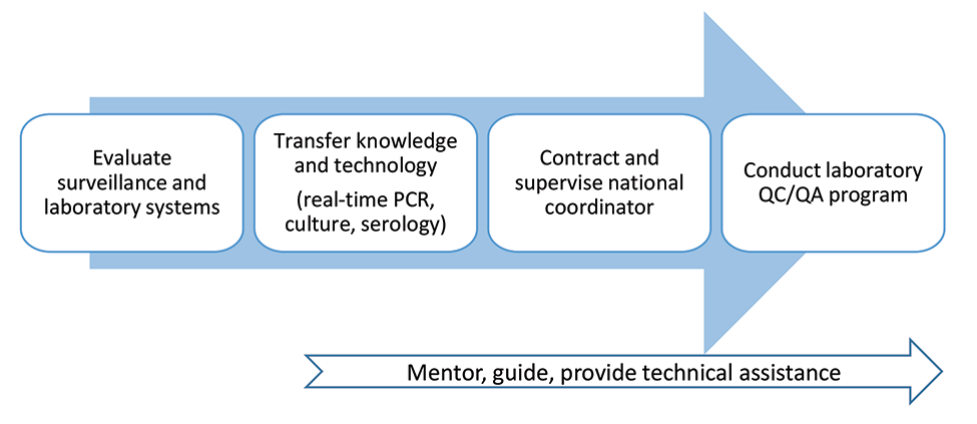
Latin American Pertussis Project model to strengthen pertussis surveillance, currently in use in 6 countries in Latin America. QC/QA, quality control and quality assurance.

The project’s specific objectives are to expand laboratory capacity for identification of *B. pertussis,* strengthen laboratory-based pertussis surveillance, and standardize and improve pertussis reporting within each country. To achieve these objectives, the LAPP strategy includes an initial in-country assessment of the pertussis surveillance system and laboratory capacity. Based on country-specific findings, each country receives on-site laboratory and epidemiologic training, guidance, and technical assistance, and participates in a laboratory quality control and quality assurance (QC/QA) program. The model used to strengthen surveillance focuses on mentoring and ongoing communication with laboratory and surveillance country staff on each specified activity (Figure 2).

LAPP goals are consistent with the objectives set by the Global Health Security Agenda (GHSA), which was launched in 2014, to strengthen global and national capacity to respond to infectious disease threats (*26**,**27*). LAPP activities support GHSA goals by improving national reference laboratory diagnostic capacity and strengthening national MOH surveillance and reporting through training and ongoing mentoring (*26*,*27*). Many LAPP country partners are also GHSA members.

Countries were selected for inclusion in LAPP on the basis of reported pertussis disease burden, potential for integrating and sustaining new laboratory capacities, and country-level requests to PAHO and CDC for technical support. LAPP began in 2009 with 3 participating countries, Argentina, Mexico, and Panama, and expanded to include Chile and Colombia in 2013 and Brazil in 2015. Budget restrictions limited extension of LAPP beyond these 6 countries. 

## Initial LAPP Surveillance Evaluation

In all participating countries, a 1- to 2-week in-country evaluation of the national surveillance system is conducted by a LAPP technical team comprising epidemiology and laboratory staff from CDC, the PAHO regional advisor for immunization, and, in many countries, the in-country PAHO representative (Table). In each country, the LAPP technical team works directly with the central-level MOH personnel from both the NRL and pertussis surveillance programs. Procedures from CDC’s Updated Guidelines for Evaluating Public Health Surveillance Systems and a standardized laboratory questionnaire are used to evaluate the pertussis surveillance system (*28*). Activities include interviews of key stakeholders and review of data sources at national, state, and local levels. To provide an overview of national pertussis surveillance function, each MOH selects >3 sites to visit; these sites are representative of different levels of surveillance reporting and performance within the country and may include hospitals and health facilities in addition to local and regional health departments.

**Table:** LAPP activities and participation for pertussis epidemiology in 6 countries in Latin America, with evaluation dates*

Country	Dates of surveillance evaluation	Laboratory diagnostics training†	Participate in LAPP QC/QA	Epidemiologic surveillance training	Epi Info training	Provider awareness training
Argentina	2009 Nov–Dec	Y	Y	Y	Y	N
Panama	2010 Jan	Y	Y	Y	Y	Y
Mexico	2010 Nov	Y	Y	Y	N	N
Chile	2013 Jan–Feb	Y	Y	N	N	N
Colombia	2013 July–Aug	Y	Y	N	Y	Y
Brazil	2015 May	Y	Y	N	Y	N

*LAPP, Latin America Pertussis Project; QC/QA, quality control and quality assurance †Training included culture and multitarget real-time PCR for all countries, and included serology for Argentina, Mexico, Chile, Colombia, and Brazil.

The LAPP technical team meets with national, regional, and local officials who share information on pertussis surveillance system organization and reporting, pertussis vaccination schedule and coverage, outbreak investigation data, laboratory diagnostic capacity and network organization, and data management. Semistructured interviews are conducted to identify system strengths, weaknesses, and areas where LAPP assistance could reinforce surveillance.

To evaluate laboratory capacity for detecting *B. pertussis,* the LAPP technical team visits the NRL and regional laboratories to comprehend workflow and assess availability of appropriate equipment and space for testing and results analysis. The team uses a structured questionnaire to obtain information about laboratory procedures, specimen collection and transportation, diagnostic assay protocols, data entry and management, and biosafety.

At the conclusion of the in-country assessment, the LAPP technical team provides the MOH with a detailed written report that summarizes potential opportunities and challenges and recommends activities to strengthen pertussis surveillance. After the assessment, in collaboration with the MOH, the team develops a work plan that prioritizes activities in both laboratory diagnostics and epidemiology surveillance.

## Expanding In-Country Laboratory Capacity for Identification of *B. pertussis*

Laboratory capacity for identification of *B. pertussis* is expanded through training in complementary pertussis diagnostics. Based on the in-country assessment and country interest, the LAPP technical team provides laboratory training for NRL staff in >2 pertussis diagnostics: nasopharyngeal culture, multitarget real-time PCR, or single-point anti–pertussis toxin IgG serology. The 1-week, in-country laboratory training course reviews pertussis diagnostics; natural history of disease; optimal testing schedules; and specimen collection, transport, processing, and storage. Training also provides hands-on practice using the 3 diagnostic tests, including assay documentation, reviewing QC/QA test criteria, troubleshooting, interpretation of results, and reporting. Training on pertussis culture focuses on appropriate sample collection and use of a biochemical testing algorithm to distinguish *Bordetella* species. Countries are trained in use of a multitarget, real-time PCR assay, which is known to be sensitive and specific, and enables identification of multiple *Bordetella* species that can cause pertussis-like disease (*29*). The serologic assay, developed by CDC in collaboration with the US Food and Drug Administration (FDA), is a highly specific, quantitative, single-point, and reference-calibrated ELISA that requires minimal reagent preparation and temperature control (*19*). Laboratory training details the advantages and limitations of each assay and emphasizes the importance of a comprehensive diagnostic program that encompasses the 3 complementary diagnostic tests. LAPP donates reagents and materials for the training and provides technical support to the NRL through email/telephone communication and in-country follow-up visits. On the basis of the individual situation of each country, LAPP may donate a real-time PCR instrument, a temporary supply of reagents, or both to ensure implementation of the new diagnostic tests. All 6 participating LAPP countries received laboratory training in pertussis culture and multitarget real-time PCR, and 5 received training on single-point IgG serologic assays. LAPP donated a real-time PCR instrument to the NRL in Argentina, Brazil, Colombia, and Panama.

## Strengthening Laboratory-Based Pertussis Surveillance

After the laboratory training, LAPP provides continued strengthening of pertussis laboratory surveillance through ongoing technical support for NRL staff in diagnostic testing, and assistance with implementation of a QC/QA program. The LAPP technical team provides mentoring of trained staff through quarterly teleconferences, 1–3 follow-up visits per country, and frequent correspondence regarding diagnostic issues as they arise (Figure 2). If requested, additional technical guidance on other methods, such as molecular typing, pulsed-field gel electrophoresis, and pertactin deficiency screening, are provided through sharing of standard operating procedures. In addition, LAPP encourages country partners to provide ongoing training on specimen collection and transport at the local level to support improved surveillance.

LAPP encourages the implementation of QC/QA measures, which are crucial to the reliability of laboratory results. LAPP supports NRLs with an annual QC/QA testing program by sending panels of blinded specimens for multitarget real-time PCR testing. Depending on the interest of each country, QC/QA panels can also be shared for pertussis culture and serology. Panel concordance with CDC results is assessed, results are shared with the country, and overall performance is shared with all partners. Currently, all 6 LAPP countries are participating in the multitarget real-time PCR QC/QA program; 1 country participates only in culture QC/QA and 1 only in serology QC/QA.

## Standardizing and Improving Pertussis Reporting within Each Country

To better understand the true burden of pertussis disease in the region, LAPP works with national-level MOH staff to ensure and strengthen standardized case reporting procedures and regularly monitor and analyze surveillance data. Specific LAPP activities depend on the unique situation and interest of each country. Examples of these activities include review of case definitions, standardization of national reporting forms, and development of surveillance indicators. In many Latin America countries, surveillance coordinators are charged with oversight of multiple vaccine-preventable diseases, limiting their ability to focus on pertussis and strengthen associated surveillance activities. Therefore, to ensure improvements in surveillance data quality, LAPP may employ a national pertussis surveillance coordinator, who is co-managed by LAPP and the MOH. National pertussis coordinators were hired by LAPP in Argentina, Panama, and Brazil.

LAPP supports improvement of pertussis reporting by providing additional training as funding allows. Staff from Argentina, Mexico, and Panama participated in a 3-week training course on epidemiology, surveillance, and data analysis, which was held at CDC in November 2011. Surveillance coordinators receive ongoing epidemiology technical assistance through biweekly to quarterly teleconferences with the LAPP technical team and in-country training, as needed. For example, in Argentina, Brazil, Colombia, and Panama, LAPP provided in-country training on Epi Info (https://www.cdc.gov/epiinfo), a free and publicly available suite of epidemiology software tools provided by CDC, to facilitate data management and analysis for surveillance reporting. In response to a country’s request for assistance amid increasing suspicion and detection of pertussis among medical and public health providers, LAPP developed and presented an in-country provider awareness training that reviewed pertussis epidemiology and clinical presentation, along with best practices for diagnosis, treatment, and laboratory diagnostics.

In an effort to understand differences in surveillance and reporting in each country, and to foster collaboration and communication in the region, LAPP hosts quarterly teleconferences to facilitate communication on topics of the participants’ choosing. Initially, each participating country gives a presentation on its surveillance system, providing an opportunity for colleagues to discuss different approaches to disease identification and reporting. Examples of other teleconference topics include pertussis case definitions, laboratory testing and capacity, chemoprophylaxis recommendations, antimicrobial drug resistance, and infant immune response after maternal pertussis vaccination. These activities support GHSA priorities to improve disease detection by promoting communication between reference laboratories and surveillance staff, and among countries participating in LAPP.

As new prevention and control strategies are introduced, country emphasis may shift from strengthening surveillance activities to evaluating the effectiveness of specific vaccine policies or interventions. LAPP is able to adjust its role and act as a mentor for these special studies by providing feedback on study methods and data analyses (*30*,*31*). Many Latin America countries have recommended pertussis vaccination during pregnancy to decrease the risk for disease among infants (*6**,**9*), and LAPP has provided methodological support for country-specific studies to assess the impact of this strategy. For these special studies, the MOH and local sites participating in these activities are responsible for seeking in-country internal review board and ethics approvals.

## LAPP Lessons Learned and Next Steps

During the initial 6 years of LAPP, several common themes have emerged, providing valuable lessons and influencing the program’s planned next steps. Upon joining LAPP, all countries were committed to strengthening their pertussis culture techniques and expanding their pertussis laboratory capacity to include functions, such as multiple real-time PCR targets and serology. Although all NRLs have incorporated real-time PCR into their diagnostic assessment for pertussis, many partners faced challenges in securing funding to support these diagnostics or in finding local providers of reagents and supplies, even when adequate funding was available. LAPP faced similar challenges in sending donated equipment, reagents, and related supplies, because of the complicated importation processes that exist in many countries. National commitment is essential for sustainability of these new diagnostics, which will depend on identifying funding and providers for equipment, reagents, and related supplies.

Another lesson learned is that ongoing mentorship and communication are key components in the process of strengthening existing surveillance systems. Successful transfer of new laboratory diagnostic processes and assistance in standardizing disease reporting required continuous support and discussion among dedicated partners. Regular teleconferences, including the LAPP technical team and individual or multiple countries, provided the opportunity to troubleshoot issues such as diagnostic implementation or analysis of surveillance data. In addition, adding the position of surveillance coordinator to concentrate on pertussis surveillance facilitated the communication process between LAPP and MOH and among in-country laboratory and surveillance staff.

LAPP has promoted communication and collaboration between LAPP-associated countries through regularly scheduled teleconferences for both laboratory and surveillance staff. These teleconferences have served as a forum for sharing country experiences and lessons learned, as well as discussing topics of interest and proposed collaborations. Ongoing communication can lead to new opportunities; discussion among partners has led to requests for country-specific trainings. For example, Argentina, Brazil, Colombia, and Panama requested Epi Info training to facilitate analysis of their surveillance data. In addition, such communication and collaboration between countries could facilitate the ability to respond rapidly to outbreaks across country borders and harmonize the regional response.

Although LAPP has established itself operationally, further work remains. Known challenges should be addressed to sustain improvements in pertussis surveillance. Key among these will be assisting LAPP countries in identifying sources and funding for diagnostic reagents and other needed supplies. Equally necessary is demonstrating the effects of LAPP activities on pertussis surveillance at the country level. Evidence of success is essential for project sustainability and will encourage participating countries to continue to improve their pertussis surveillance programs. Evidence of success may lead to additional funding sources, which would allow LAPP to expand the partnership to other interested countries. Finally, LAPP could continue to provide technical mentorship for special studies as new regionally or globally relevant research questions arise.

LAPP supports strategies endorsed by multiple public health entities, such as the WHO Global Vaccine Action Plan (*32*), PAHO Regional Immunization Vision and Strategy (*33*,*34*), and GHSA. Specifically, LAPP activities that increase laboratory capacity to detect disease help inform immunization policy and support the GHSA goals to detect, characterize, and report potential outbreaks early. LAPP’s focus on providing laboratory diagnostic and epidemiology training also aligns with the GHSA priority on training an effective biosurveillance workforce; the surveillance skills obtained through LAPP training may be transferable to other disease threats. 

In conclusion, LAPP has developed a partnership between CDC, PAHO, Sabin, and the MOHs of 6 Latin America countries to strengthen national laboratory and surveillance capacity to more rapidly and accurately detect and monitor pertussis. Such efforts can contribute to more rapid control of pertussis outbreaks and thereby enhance global health security. Subsequent areas of emphasis include demonstrating the effect of LAPP activities at the country level; continuing to address the challenges partners face in sustaining these improvements; focusing efforts to expand laboratory-based surveillance for pertussis to other Latin American countries; and continuing to support special studies to answer relevant research questions.
